# Exploring the potential of intranasally administered naturally occurring quercetin loaded into polymeric nanocapsules as a novel platform for the treatment of anxiety

**DOI:** 10.1038/s41598-023-27665-6

**Published:** 2023-01-10

**Authors:** Khaled Y. Mahmoud, Nahla A. Elhesaisy, Abdelrahman R. Rashed, Ebram S. Mikhael, Mahmoud I. Fadl, Mahmoud S. Elsadek, Merna A. Mohamed, Merna A. Mostafa, Mohamed A. Hassan, Omar M. Halema, Youssef H. Elnemer, Shady A. Swidan

**Affiliations:** 1 Department of Pharmaceutics and Pharmaceutical Technology, Faculty of Pharmacy, The British University in Egypt, El-Sherouk City, 11837 Cairo Egypt; 2 Faculty of Pharmacy, The British University in Egypt, El-Sherouk City, 11837 Cairo Egypt

**Keywords:** Diseases, Nanoscience and technology

## Abstract

Anxiety is one of the most prevalent forms of psychopathology that affects millions worldwide. It gained more importance under the pandemic status that resulted in higher anxiety prevalence. Anxiolytic drugs such as benzodiazepines have an unfavorable risk/benefit ratio resulting in a shift toward active ingredients with better safety profile such as the naturally occurring quercetin (QRC). The delivery of QRC is hampered by its low water solubility and low bioavailability. The potential to enhance QRC delivery to the brain utilizing polymeric nanocapsules administered intranasally is investigated in the current study. Polymeric nanocapsules were prepared utilizing the nanoprecipitation technique. The best formula displayed a particle size of 227.8 ± 11.9 nm, polydispersity index of 0.466 ± 0.023, zeta potential of − 17.5 ± 0.01 mV, and encapsulation efficiency % of 92.5 ± 1.9%. In vitro release of QRC loaded polymeric nanocapsules exhibited a biphasic release with an initial burst release followed by a sustained release pattern. Behavioral testing demonstrated the superiority of QRC loaded polymeric nanocapsules administered intranasally compared to QRC dispersion administered both orally and intranasally. The prepared QRC loaded polymeric nanocapsules also demonstrated good safety profile with high tolerability.

## Introduction

Anxiety is defined by the American Psychological Association (APA) as “an emotion characterized by feelings of tension, worried thoughts and physical changes like increased blood pressure”^1^. The Global Burden of Diseases, Injuries, and Risk Factors Study (GBD) 2019 identified anxiety disorders as the most prominent mental disorder. This high prevalence was present for both sexes, and across the entire lifespan^2^. World struck Covid-19 pandemic resulted in intensifying the already high prevalence of anxiety. Daily infection rates and human mobility reductions were associated with increased anxiety prevalence^3^.

Anxiety disorders are characterized by enduring and excessive fear, and avoidance of perceived threats such as social situations or body sensations. Also, panic attacks can manifest as a form of abrupt fear response^4^. Anxiety disorders can be classified into specific phobia, generalized anxiety disorder, social anxiety disorder, agoraphobia, and panic disorder^5^. Multiple treatment options for anxiety disorders including azapirones, benzodiazepines, serotonin norepinephrine reuptake inhibitors, antipsychotics, antihistamines, and alpha- and beta-adrenergic medications^6^.

Despite the presence of multiple classes of anxiety treatment, full disease treatment is still un-achievable. Conventional pharmacological approaches are characterized by multiple side effects and poor tolerability profile^7^. The need for active ingredients with higher safety profile promoted a shift toward phytochemicals. Phytochemicals are low molecular weight secondary metabolites that exist naturally in plants^8^. Among the different phytochemicals present, Quercetin (QRC) was reported as an anxiolytic^9^.

QRC (2-(3,4-dihydroxyphenyl)-3,5,7-trihydroxychromen-4-one) is one of the most common flavonoids of polyphenols present in many plants such as red onions and kale^10^. QRC has a large spectrum of reported pharmacological actions ranging from anti-diabetic effects to anti-cancer effects^11^ aside from its reported anxiolytic effect. Multiple articles reported the effects of QRC on anxiety. These articles are summarized in a previously published review article by Silvestro et al.^12^.

Different mechanisms have been reported previously for the anxiolytic effects of QRC. One of these mechanisms is the chelation of iron overload in the brain. It has been reported that 3-hydroxyl and 4-carbonyl groups of QRC are the potential iron chelating sites^13–15^. Other reported mechanisms include the modulation of corticosterone, γ-aminobutyric acid (GABA), and serotonin^16,17^.

Despite the huge benefits of QRC, its use is hampered by its low water solubility and low oral bioavailability^18^. One of the most common approaches to overcome these limitations is the utilization of nanoparticles. Nanoparticles are defined as 3D objects with 3 external dimensions in the nano range with the nano range ranging from 1 and 1000 nm^19^.

QRC have been previously incorporated in different lipid based nanoparticles for the treatment of anxiety and anxiety related disorders^17,20–22^. Despite the advantages of the prepared formulations, lipid based nanoparticles possess some limitations that may hamper their effective usage including short half-life, risk of oxidation and hydrolysis of the used phospholipid and risk of drug expulsion^23^.

Among the different classes of nanoparticles, polymeric nanoparticles have generated great interest because of their advantages. These advantages including controlled drug release, biocompatibility, biodegradability, and drug protection against the environment^24^. Polymeric nanoparticles can be classified into nanospheres and nanocapsules. Nanospheres compose of homogenous solid polymeric matrix while nanocapsules consist of liquid/solid core surrounded by a polymeric shell. Over recent years, polymeric nanocapsules attracted significant interest in the drug delivery field. This interest can be attributed to the higher drug loading capacity and lower polymer content compared to polymeric nanospheres^25^.

Among the various available routes of administration, intranasal route is the preferred route of administering active pharmaceutical ingredients directly to the brain^26^. The intranasal pathway allows for either delivery of therapeutics directly to the brain from the nose bypassing the blood–brain barrier (BBB) by traveling extracellularly along the olfactory and trigeminal nerve pathways, or through the vasculature route followed by system circulation in which passing the BBB is required^27^. Nanoparticles possess multiple advantages rendering them ideal candidates for intranasal delivery such as thermodynamic stability, and the ability to penetrate biological membranes including the BBB^28,29^.

Despite the high prevalence of anxiety, concurrently higher disease burden, there have been far lower number of recent research on novel anxiety treatments compared to number of experimental drugs for other disorders such as major depressive disorder, and schizophrenia^6^. The aim of the present study is to prepare and evaluate for the first time -to the best of our knowledge- QRC loaded polymeric nanocapsules as an anxiety treatment utilizing the intranasal route. The effect of amount of drug and the type of surfactant on the polymeric nanocapsules characteristics was evaluated and in vivo behavioral assessments were conducted.

## Materials and methods

### Materials

QRC (≥ 95%), Polycaprolactone (PCL)-Mwt. 14000, and Benzalkonium Chloride were purchased from Sigma-Aldrich (St. Louis, MO, USA). Tween 80, and Poloxamer 188 (Pluronic F68) were purchased from Alfa Aesar (Lancashire, United Kingdom). Span 80 (Sorbitan Mono-oleate) was purchased from Oxford Laboratory Chemicals (Maharashtra, India). Capryol 90 (Propylene glycol monocaprylate) was a kind gift from Gattefosse (Saint-Priest, Cedex, France). Acetone analytical grade was purchased from Piochem (Giza, Egypt). All chemicals were used as received without modifications.

### Theoretical screening of QRC solubility in different liquid lipids

Hansen Solubility Parameters (HSP) were used to theoretically predict the miscibility between QRC and twelve different liquid lipids namely Capryol 90, Capryol PGMC, Captex 200P, Captex 355, Ethyl Oleate, Isopropyl Myristate, Labrafil M 1944 CS, Labrafil M 2125 CS, Labrafac Lipophile WL 1349, Labrafac PG, Miglyol 810, and Oleic Acid. HSP of QRC and different liquid lipids were calculated using version 5.3.06 Hansen Solubility Parameters in Practice (HSPiP) software (Hansen Solubility, Hørsholm, Denmark) employing Hiroshi Yamamoto’s molecular breaking method (Y-MB). The chemical structures of different materials were obtained using ChemBioDraw Ultra version 14.0 (CambridgeSoft corporation, Cambridge, MA, USA) through utilizing the IUPAC name of each material from PubChem database (available from https://pubchem.ncbi.nlm.nih.gov/). Each structure was converted by ChemBioDraw Ultra software to its simplified molecular input line entry syntax (SMILES) notation to be fed to HSPiP software generating the different solubility parameters in silico. The solubility parameters obtained were δ_D_ which refers to dispersion forces (van der Waals), δ_P_ that refers dipole forces (polarity), and δ_H_ which refers to hydrogen bonding. Euclidean distance [Eq. (1)] was used to predict the solubility between QRC and different lipids where solute refers to QRC and solvent refers to the lipids^30^.

1 $$\mathrm{D}=\sqrt{{{(\delta}{\text{D}}\left({\text{solute}}\right)\text{- }\delta \text{D}\left({\text{solvent}}\right)\text{)}}^{2}\text{ + }{\text{(}\delta \text{P}\left({\text{solute}}\right)\text{- } \delta \text{P}\left({\text{solvent}}\right)\text{)}}^{2}\text{ + }{\text{(} \delta \text{H}\left({\text{solute}}\right)\text{- } \delta \text{H}\left({\text{solvent}}\right)\text{)}}^{2}}$$

### Solubility study

QRC solubility in Capryol 90 was determined using a modified shake method^31^. Excess amount of QRC was added to one gram of Capryol 90 in a glass vial. The prepared dispersion was placed in a shaking water bath (Sci Finetech Shaking Water Bath, Korea) set at 25 ± 0.5 °C and 50 RPM for 24 h. The undissolved QRC was removed through centrifugation for 1 h at 12,000 RPM and 25 °C (PRO-Research K241R; Centurion, West Sussex, United Kingdom). the obtained supernatant was appropriately diluted and measured spectrophotometrically at λ_max_ = 367 nm using UV spectrophotometer (Jenway 6305 spectrophotometer, China).

### Preparation of QRC loaded polymeric nanocapsules

Polymeric nanocapsules entrapping QRC were prepared using a modified nanoprecipitation method^32^. QRC, PCL (100 mg), span 80 (40 mg), and Capryol 90 (90 μl) were dissolved together in 25 ml acetone to prepare the organic phase. The aqueous phase was prepared by dissolving the surfactant in 50 ml distilled water. The prepared organic phase was added drop wise onto the aqueous phase under moderate stirring to allow for the formation of nanocapsules. The prepared nano-dispersion was added into a round bottom flask placed in a rotary evaporator (SCILOGEX Rotary Evaporator Rotavapor RE100-Pro; SCILOGEX, Rocky Hill, CT, USA) set at 40 °C under reduced pressure to allow for the evaporation of acetone as well as concentrating the prepared dispersion to 25 ml.

2^2^ full factorial design was employed to assess the effects of changing QRC amounts and the type of surfactant used on particle size, zeta potential, and entrapment efficiency. Two different surfactants were used namely tween 80 and poloxamer 188 with two different QRC amounts which are 10 mg and 30 mg. This resulted in four different formulations stated in Table 1.

**Table 1:** Different polymeric nanocapsules formulations prepared.

Formula	QRC (mg)	Tween 80 (mg)	Poloxamer 188 (mg)
F1	10	80	–
F2	10	–	80
F3	30	80	–
F4	30	–	80

### QRC loaded polymeric nanocapsules characterization

#### Particle size, polydispersity index (PDI) and zeta potential

Dynamic light scattering was employed to determine particle size, polydispersity index and zeta potential for all the prepared formulation employing Malvern Zetasizer (Malvern Instruments, Malvern, UK) at 25 °C. One ml of each formulation was diluted to a total of 10 ml using distilled water before measurements.

#### Encapsulation efficiency (EE)%

The amount of QRC encapsulated in all the prepared formulation were determined indirectly through analyzing the amount of unencapsulated drug^33^. Two ml of each formula was centrifuged at 15,000 RPM for 2 h at 4 °C using cooling centrifuge. 100 μl of supernatant was diluted to a total of 800 μl using 20% ethanol in deionized water solution and measured spectrophotometrically at λ_max_ = 367 nm using UV spectrophotometer. EE% was calculated using the following equation:

$$\text{EE}\%= \frac{{\text{W}}_{\text{initial}}-{\text{W}}_{\text{free}}}{{\text{W}}_{\text{initial}}}$$

where W_initial_ is the initial amount of the drug used and W_free_ is the amount of drug present in the supernatant^34^.

Based on the previous characterization techniques, the best formulation will be chosen. The following characterization techniques are done for the chosen formulation.

#### Transmission electron microscopy (TEM)

Morphological evaluation of the chosen formula was evaluated using TEM (JTEM-1010, JEOL, Tokyo, Japan) utilizing negative staining technique. One drop of the chosen formula was placed on a carbon film covered copper grid. Uranyl acetate solution (2% w/v) was added drop wise onto the grid followed by sample drying by air at room temperature. TEM investigations were done at 80 kV.

#### Differential scanning calorimetry (DSC)

Thermal behavior of QRC, PCL, Capryol 90, physical mixture of QRC, PCL and Capryol 90 in the ratio used in the preparation of polymeric nanocapsules which is 3:10:9 and the chosen formula were analyzed using DSC (DSC-50; Shimadzu, Kyoto, Japan). First, the chosen formula was lyophilized using freeze dryer (Alpha 1-2LD plus; Christ, Osterode am Harz, Germany) at − 45° C for 14 h. Second, accurately weighed amount of each sample was sealed in an aluminum pan. The pan was heated from 30 to 400 °C with a rate of 10 °C/min under nitrogen atmosphere. The data were analyzed using TA-50 WSI thermal analyzer (Shimadzu, Kyoto, Japan).

#### X-Ray diffraction (XRD)

Crystalline or amorphous properties of QRC, PCL, physical mixture of QRC and PCL in the ratio used in the preparation of polymeric nanocapsules which is 3:10 and the chosen formula were evaluated using X-ray diffractometer (D8; Bruker Co., Germany). The scanned diffraction angle 2θ ranged from 5° to 90° with a scan rate of 1°/min.

### In vitro release study

#### In vitro release of unencapsulated QRC and QRC loaded polymeric nanocapsules

Dialysis bag technique was used to evaluate the in vitro release of the chosen QRC loaded polymeric nanocapsules formulation and compare it to QRC dispersion in deionized water. One ml of the chosen formula and one ml of QRC dispersion containing equivalent amount of QRC were placed in tightly sealed dialysis bag (molecular weight cutoff 12,000–14,000). The dialysis bags were placed in 100 ml beakers comprising 50 ml phosphate buffer saline pH 7.4 containing 1% tween 80 as the release media. The beakers were placed in a shaking water bath set at 37 ± 0.5 °C and 50 RPM for 48 h. One ml sample of the release media was withdrawn at 0.5, 1, 2, 3, 4, 5, 6, 24, and 48 h. The withdrawn volume was compensated with fresh release media maintaining the same final volume and the sink condition through the entire experiment. The withdrawn samples were analyzed spectrophotometrically at λ_max_ = 367 nm. The obtained absorbances were used to calculate cumulative QRC release percentage.

#### In vitro release kinetics

Release kinetics of free QRC and QRC loaded polymeric nanocapsules was analyzed through fitting the in vitro release data onto different mathematical models namely zero order, first order, Higuchi, and Hixson–Crowell. In order to identify QRC release mechanism from the polymeric nanocapsules, Korsmeyer–Peppas model was employed. The release kinetics were obtained using DDSolver software, a menu-driven add-in program for Microsoft Excel. After fitting the experimental data, adjusted r^2^ was used as a model selection criterion.

### Animal studies

#### Animals

Adult female Sprague Dawley rats (n = 20, weight 250–310 gm, 11 weeks old) bred at the Faculty of Pharmacy, the British University in Egypt animal house were caged at standard plastic cages (5 per cage). The rats were kept under standard conditions of temperature (25 ± 0.5 °C), relative humidity (55 ± 1%) and light cycle (12 h light and 12 h dark) with free access to food and water. Dedicated efforts were made to minimize animal suffering. All animal work was carried out according to the National Institutes of Health guide for the care and use of laboratory animals and approved by the Ethical Committee of Faculty of Pharmacy, the British University in Egypt with the ethical approval number: EX-2209. The methods are reported in accordance with Arrive guidelines.

#### Experimental design

The experiment is a single dose, one-way study design. Rats were simply randomized into four groups (n = 5); control group administered saline intranasally, oral QRC dispersion, intranasal QRC dispersion, intranasal QRC loaded polymeric nanocapsules. Oral QRC dispersion group received QRC dispersed in deionized water. Oral dose was 50 mg/kg using oral gavage which was previously reported to exert anxiolytic effect^35^. Intranasal QRC dispersion, and intranasal QRC polymeric nanocapsules groups received 35 μl dispersion containing 0.15 mg/kg QRC. The intranasal dose was administered via a micropipette directly into the rats’ nostril.

#### Behavioral assessment

Rats’ behavior was assessed for their anxiety and cognitive effect using open field test and elevated plus-maze test after single administration. Behavioral assessment started 30 min after administering the intranasal dose and 150 min after administering the oral dose. The difference in time between the oral and intranasal route was established to allow time for the oral QRC to be absorbed through the gastrointestinal tract.

#### Open field test

Black plexiglas box (58 × 58 × 39 cm) was used to conduct the open field test. The test was conducted in the light period of the day in a silent room in the laboratory. Animals were placed in the center of the field and videotaped for 5 min. The field was wiped thoroughly using ethanol between each session. Videos were analyzed using ANY-maze video tracking software (Stoelting co., Illinois, USA) for time spent in the center of the field while having the data analyst not aware of the groups’ allocation.

#### Elevated plus-maze test

Standard black wooden apparatus, consist of four arms, elevated 49 cm of the ground was used for the elevated plus-maze test. The four arms were two opposite open arms (49 × 9 cm), and two opposite enclosed arms (49 × 9 × 30 cm) each placed perpendicularly relative to the adjacent arm forming a plus sign. The arms were connected using a center stage (10 × 10 cm). The test was conducted in the light period of the day in a silent room in the laboratory. Animals were placed on the center stage facing an enclosed arm and videotaped for 5 min. The field was wiped thoroughly using ethanol between each session. Videos were analyzed using ANY-maze video tracking software (Stoelting co., Illinois, USA) for the time spent in the open arms while having the data analyst not aware of the groups’ allocation.

### Safety studies

#### Observational nasal irritation test

During nasal administration conducted in animal studies, an observational nasal irritation test was conducted^36^. Visual observation was conducted when saline, QRC dispersion, and QRC loaded polymeric nanocapsules were administered intranasally. The animals were carefully monitored for signs of mucosal inflammation including discomfort, sneezing, and itching. The nasal mucosa irritation index reported by Elnaggar et al. was employed^37^. The index identifies four degrees of irritation according to percentage of animals showing irritation signs. These degrees are strong irritation (more than 60% of the animals), moderate irritation (from 30 to 60%), mild irritation (from 10 to 30%), and no irritation (up to 10%).

#### Histopathological examination

In order to investigate the nasal mucosa toxicity of the chosen formula, Histopathological evaluation was conducted following the procedure reported by Abou Youssef et al.^38^ with slight modification. Adult female Sprague Dawley rats (n = 6, weight 250–310 gm, 11 weeks old) were randomly distributed into 3 groups (n = 2); negative control group that received 35 μl saline, positive control group which received 35 μl of 0.2% benzalkonium chloride solution, and treatment group that received 35 μl of the chosen formula in a single nostril. Animals were sacrificed 20 min post intranasal administration and the nasal mucosa were carefully harvested and stored in 10% formalin solution. Rats were euthanized using a carbon dioxide system. Rats were placed in the carbon dioxide chamber for 3–5 min. Heartbeat and respiratory symptoms were carefully monitored after the rats’ removal from the chamber to confirm their absence. This was followed by cervical dislocation. The samples were embedded in paraffin, cut into four μm-thick sections, then stained using hematoxylin and eosin (H&E) for investigation using light microscope to detect any damage in the nasal mucosa.

### Statistical analysis

Data were analyzed by one-way analysis of variance (one-way ANOVA) followed by Tukey–Kramer post hoc statistical tests and presented as mean ± standard deviation (SD). The analysis was done using GraphPad Prism (V9) (GraphPad Software, San Diego, CA, USA). Values of P < 0.05 were considered to be statistically significant.

### Ethics approval

All institutional and national guidelines for the care and use of laboratory animals were followed. All animal work was approved by the Ethical Committee of Faculty of Pharmacy, the British University in Egypt with the ethical approval number: EX-2209.

## Results

### Theoretical screening of QRC solubility in different liquid lipids

**Figure 1:**
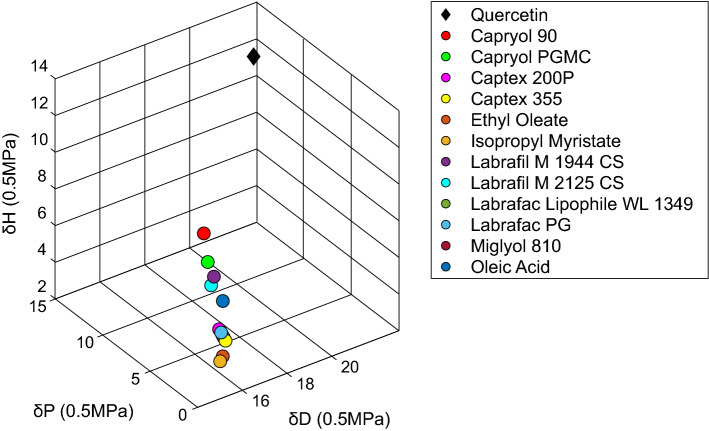
3D diagram representing the Euclidean distance between QRC and the different liquid lipids.

In order to select the best possible liquid lipid to solubilize QRC, theoretical screening was employed using HSP. The Euclidean distance between QRC and the different liquid lipids alongside the parameters of all materials are mentioned in Table 2 Each material using its parameters is plotted in a 3D diagram (Fig. 1) using Matlab version 2021a (Mathworks, Inc., Natick, MA, USA) to visualize the distance between QRC and each liquid lipid.

**Table 2:** HSP of QRC and different liquid lipids as well as the Euclidean distance between QRC and each liquid lipid.

Material	δ_D_	δ_P_	δ_H_	Euclidean distance
QRC	21	10.6	13.7	–
Capryol 90	16.39	4.98	8.41	8.99
Capryol PGMC	16.33	4.46	7.19	10.09
Labrafil M 1944 CS	16.27	3.63	6.64	10.99
Labrafil M 2125 CS	16.17	3.69	6.19	11.3
Oleic Acid	16.4	3	5.5	12.09
Captex 200P	16.23	2.99	4.04	13.19
Labrafac PG	16.25	2.83	3.92	13.36
Labrafac Lipophile WL 1349	16.42	3.06	3.58	13.43
Miglyol 810	16.43	2.99	3.51	13.51
Captex 355	16.45	2.83	3.38	13.7
Ethyl oleate	16.1	2.3	2.90	14.48
Isopropyl myristate	15.9	2.1	2.80	14.73

Among the different liquid lipids assessed, Capryol 90 exhibited the lowest Euclidean distance while isopropyl myristate showed the highest Euclidean distance.

### Solubility study

The solubility of QRC in Capryol 90 was validated experimentally. QRC demonstrated high solubility in Capryol 90 with a value of 28.41 ± 8.91 mg/g. This value was in accordance with what was previously obtained by Pangeni et al.^39^.

### QRC loaded polymeric nanocapsules characterization

Characterization is considered to be one of the critical steps in order to identify the physicochemical properties of the prepared nanoparticles^40^. The results of the prepared formulations are summarized in Table 3.

**Table 3:** Summary of the results obtained for all the prepared formulae.

Formula	Particle size (nm)	PDI	Zeta potential (mV)	EE%
F1	184.6 ± 4.4	0.19 ± 0.015	− 14.1 ± 0.14	79.1 ± 2.3
F2	262.3 ± 1.6	0.268 ± 0.017	− 15 ± 0.57	83.9 ± 1.6
F3	227.8 ± 11.9	0.466 ± 0.023	− 17.5 ± 0.01	92.5 ± 1.9
F4	278.1 ± 3.7	0.371 ± 0.018	− 18.7 ± 1.70	92.7 ± 2.1

All the prepared nanocapsules demonstrated a size in the nano scale ranging from 184.6 nm for F1 to 278.1 nm for F4. PDI values ranged from 0.19 for F1 to 0.466 for F3. These PDI values indicated good homogeneity for all of the prepared formulations as it was previously reported that the ideal PDI value is less than 0.5^41^. Regarding zeta potential, the prepared nanocapsules exhibited values ranging from − 14.1 mV for F1 to − 18.7 mV for F4. All of the prepared formulations are considered to be stable as it was previously reported that zeta potential values between − 15 mV and − 30 mV are considered ideal for nanoparticles stabilization^42^ as well as the presence of steric stabilization^43^. As for EE%, the values ranged between 79.1% for F1 to 92.7% for F4.

Based on these results, F3 was chosen to be subjected to further investigations.

**Figure 2:**
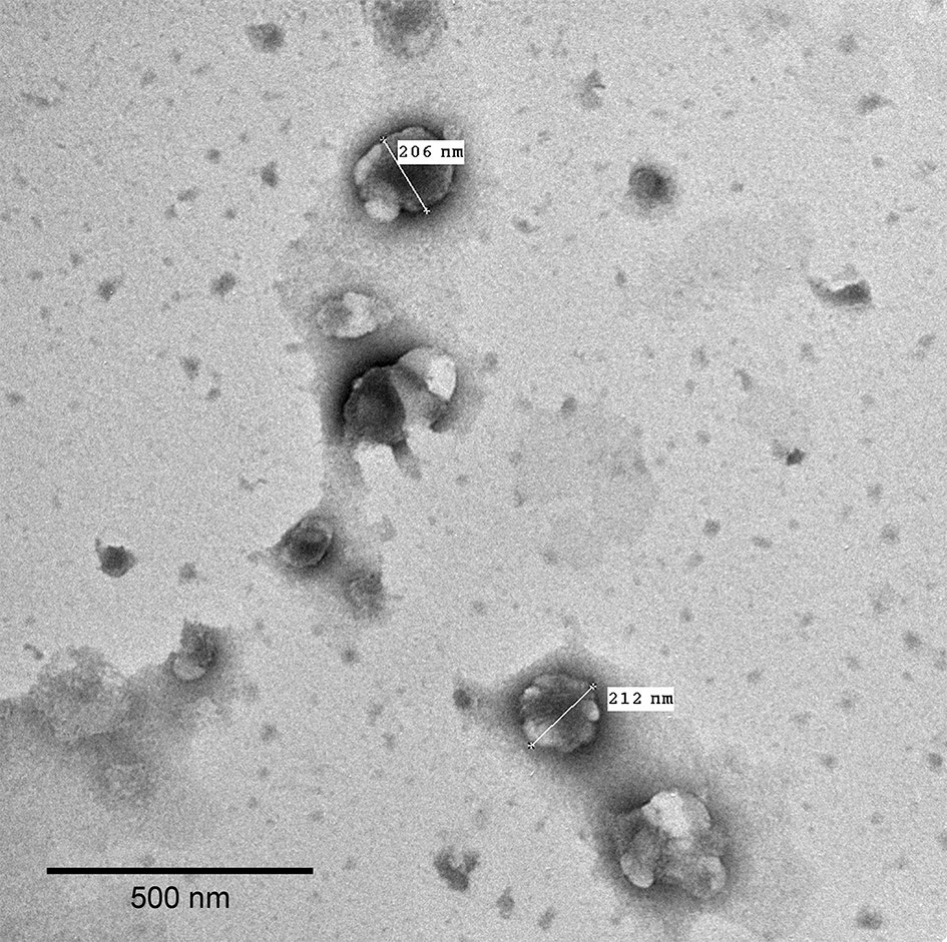
TEM image of F3.

The morphology of the chosen formula was evaluated using TEM. As shown in (Fig. 2), TEM image shows well defined, spherical shaped nanoparticles that appear to be unilamellar.

**Figure 3:**
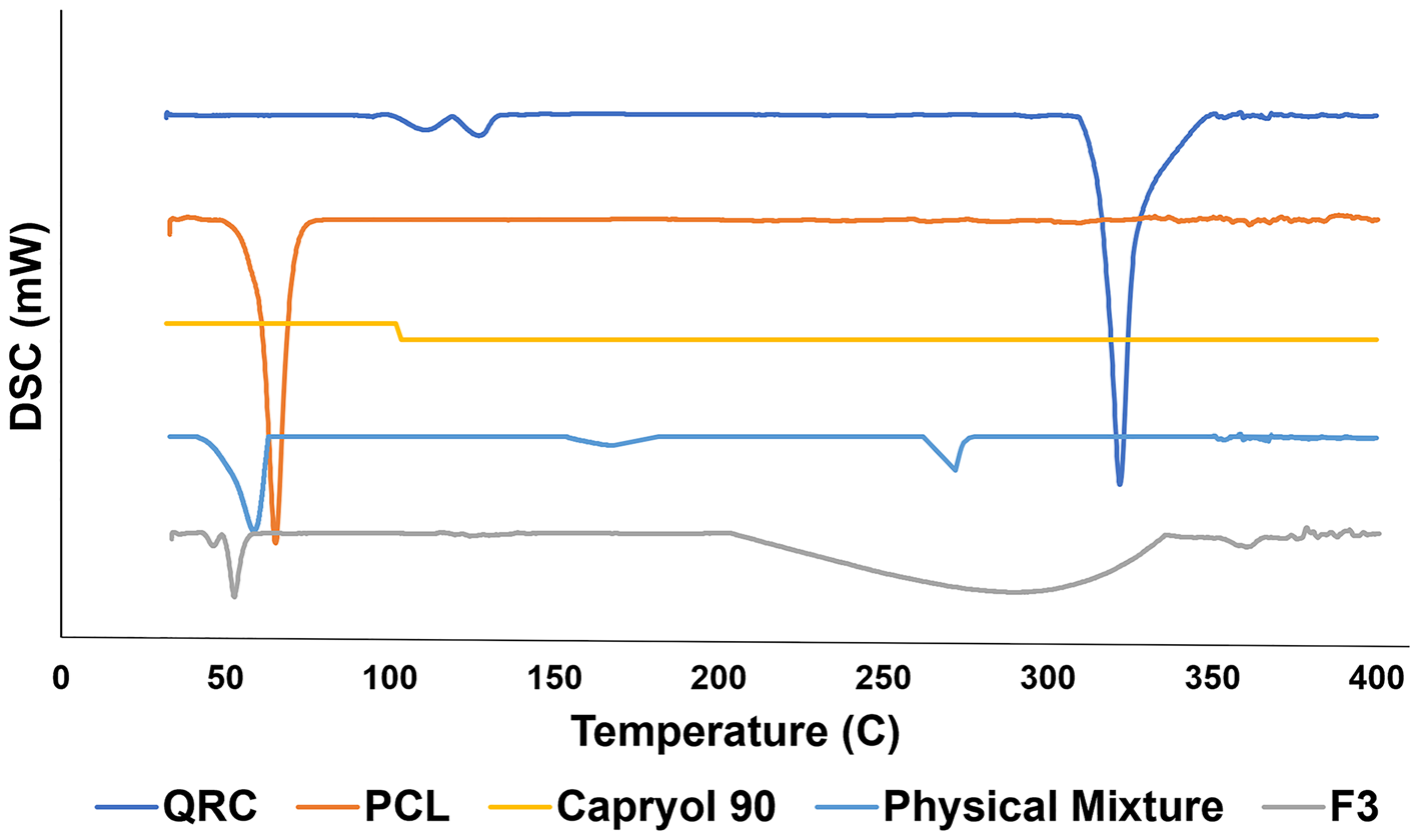
DSC charts of QRC, PCL, Capryol 90, physical mixture and F3.

Thermal analysis of QRC, PCL, Capryol 90, physical mixture, and the chosen formula using DSC (Fig. 3). QRC displayed a sharp endothermic peak at 322 °C similar to what was previously reported by Li et al.^44^. PCL displayed a sharp endothermic peak at 65.26 °C close to what was reported by González-Reza et al.^45^. Capryol 90 displayed no peaks similar to what was previously reported by Ibrahim et al.^46^. The physical mixture exhibited a shift in both melting peaks of PCL and QRC to lower melting points at 58.66 °C and 271.06 °C respectively. However, the chosen formula displayed a broad peak 290.3 °C with the disappearance of QRC peak and shifting of the PCL peak to 46.37 °C.

**Figure 4:**
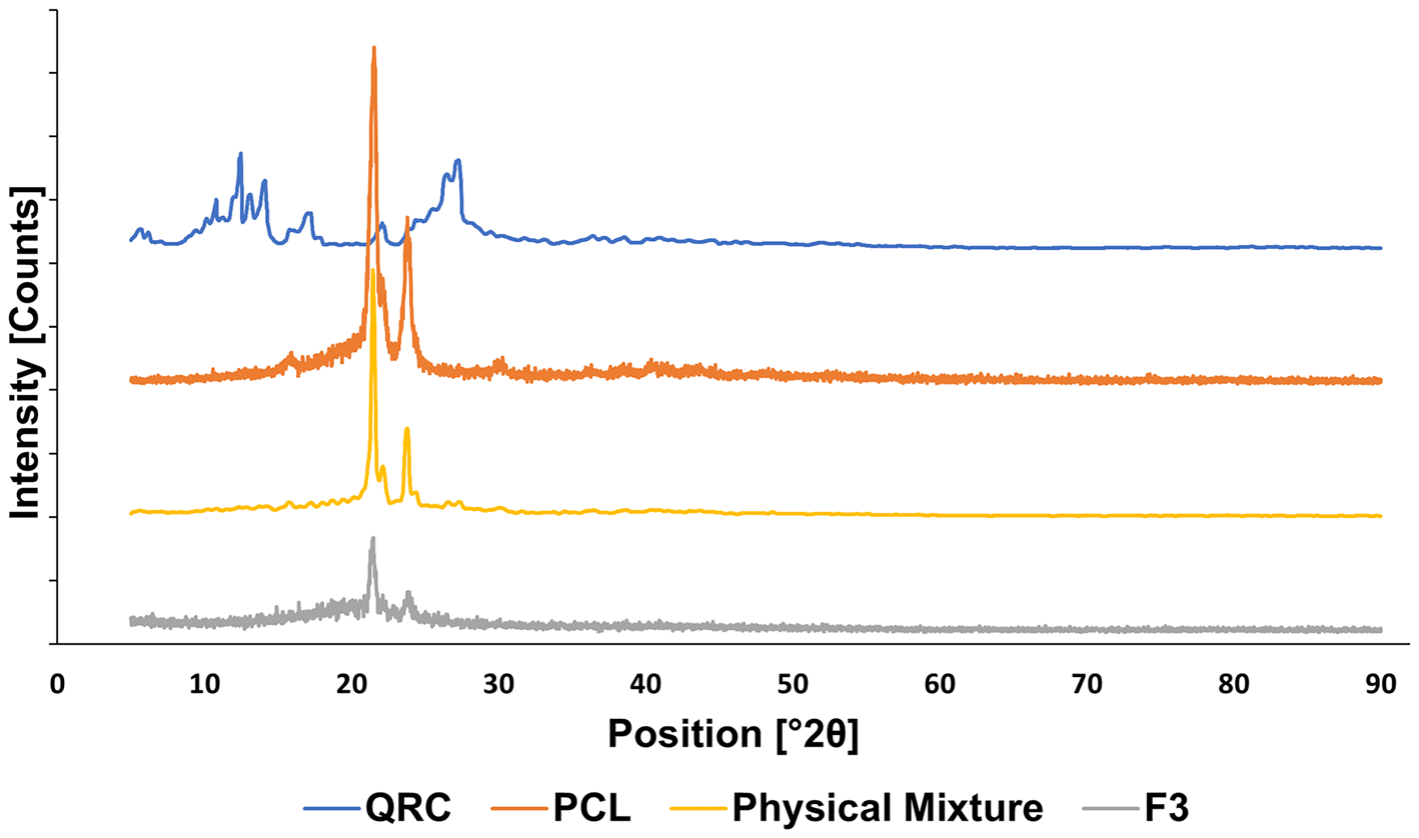
XRD charts of QRC, PCL, physical mixture, and F3.

XRD was conducted for pure QRC, PCL, physical mixture, and the chosen formula (Fig. 4). QRC displayed its highly crystalline structure^47^ through exhibiting sharp peaks at 2θ of 10.73°, 12.45°, 13.06°, 14.1°, 15.76°, 17.16°, 22.09°, 26.49°, and 26.99°. PCL displayed its semi crystalline structure^48^ by exhibiting two peaks at 2θ of 21.49°, and 23.86°. The physical mixture displayed the characteristic peaks of both PCL and QRC. Albeit the low intensity of QRC peaks which may be due to the small quantity of QRC used. The chosen formula exhibited only PCL peaks with the complete disappearance of QRC peaks indicating the drug change in structure from crystalline to amorphous.

### In vitro release study

**Figure 5:**
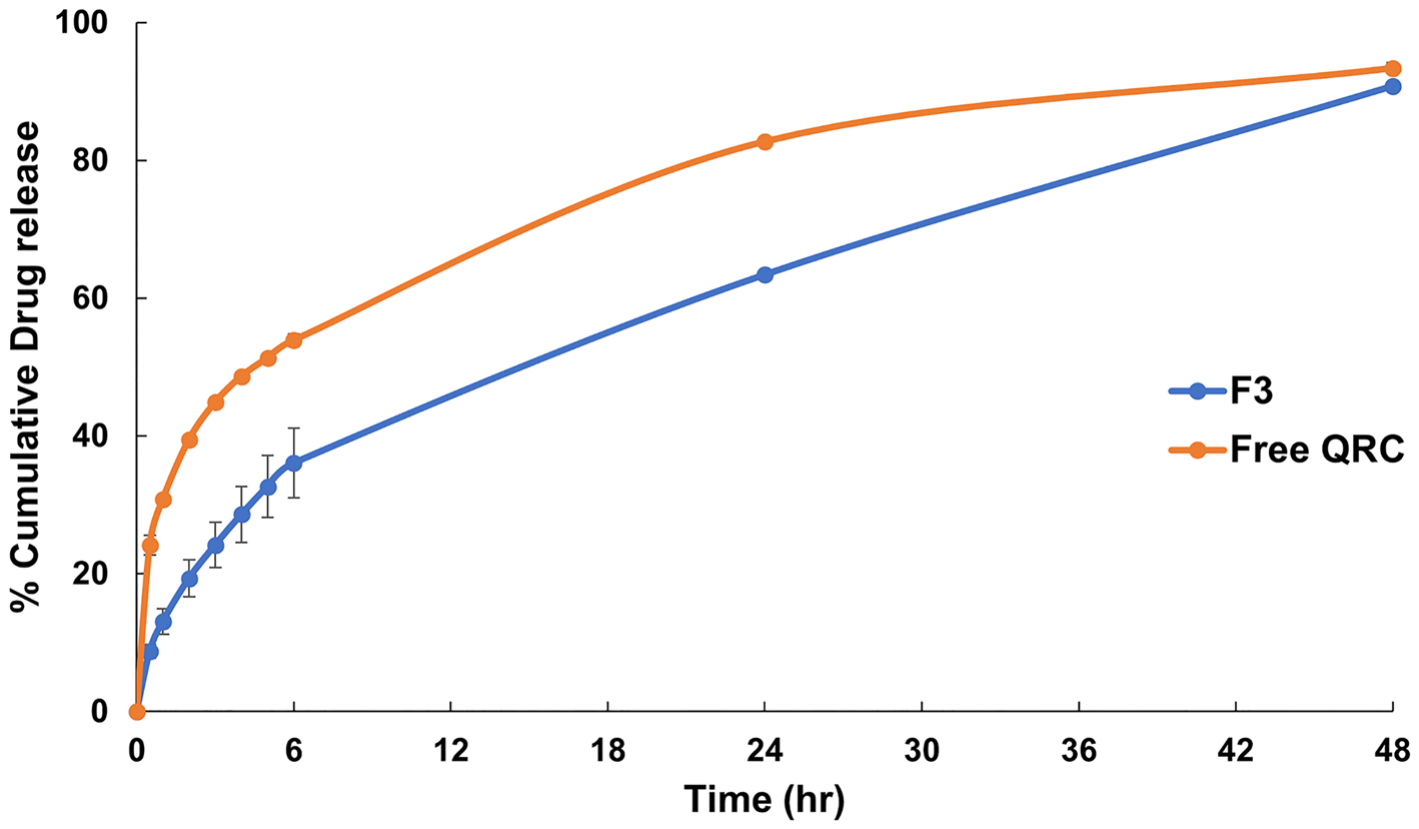
In vitro release profile of free QRC and F3.

Release pattern of both the chosen formulation of QRC loaded polymeric nanocapsules and QRC dispersion are illustrated in (Fig. 5). As shown in the figure, the prepared formulation resulted in a much slower release pattern over the course of the experiment compared to QRC dispersion. After 6 h, the cumulative percent release of the prepared nanocapsules was 36.06% compared to 53.91% of QRC dispersion. The release of the prepared nanocapsules continued to exhibit slower release after 24 h with cumulative release of 63.38% for the prepared nanocapsules compared to 82.71% for the QRC dispersion. In vitro release of QRC loaded polymeric nanocapsules exhibited a biphasic release with an initial burst release followed by a sustained release pattern with the release of both, the prepared nanocapsules and QRC dispersion, reaching almost complete release after 48 h.

Through analyzing the kinetic models applied to the release data of free QRC and QRC loaded polymeric nanocapsules (Table 4), it was possible to determine that the release profile of free QRC followed first order model (r^2^ adjusted = 0.8402). While the release profile of QRC loaded polymeric nanocapsules followed Higuchi model (r^2^ adjusted = 0.9947). The n value of Korsmeyer–Peppas model for free QRC was 0.316 with a r^2^ adjusted of 0.9983 while the n value for QRC loaded polymeric nanocapsules was 0.48 with a r^2^ adjusted of 0.9957.

**Table 4:** Summary of different release models and the corresponding r^2^ adjusted.

Model	Equation	R^2^ adjusted free QRC	R^2^ adjusted QRC loaded polymeric nanocapsules
Zero order	Q = Q_0_ + K_0_t	− 0.4732	0.3838
First order	dC/dT = − Kt	0.8402	0.7834
Higuchi	Q = K_H_ t^1/2^	0.7022	0.9947
Hixson–Crowell	Q_0_^1/3^–Q_t_^1/3^ = K_HC_ t	0.5805	0.6777

Q is the amount of drug released or dissolved, Q_0_ is the initial amount of drug in solution, t is time, K_0_ is the zero order release constant, dC is the concentration derivative, dT is the time derivative, K is the first order rate constant, K_H_ is the Higuchi dissolution rate constant, Qt is the remaining weight of solid at time t, K_HC_ is the Hixson–Crowell dissolution rate constant.

### Animal studies

**Figure 6:**
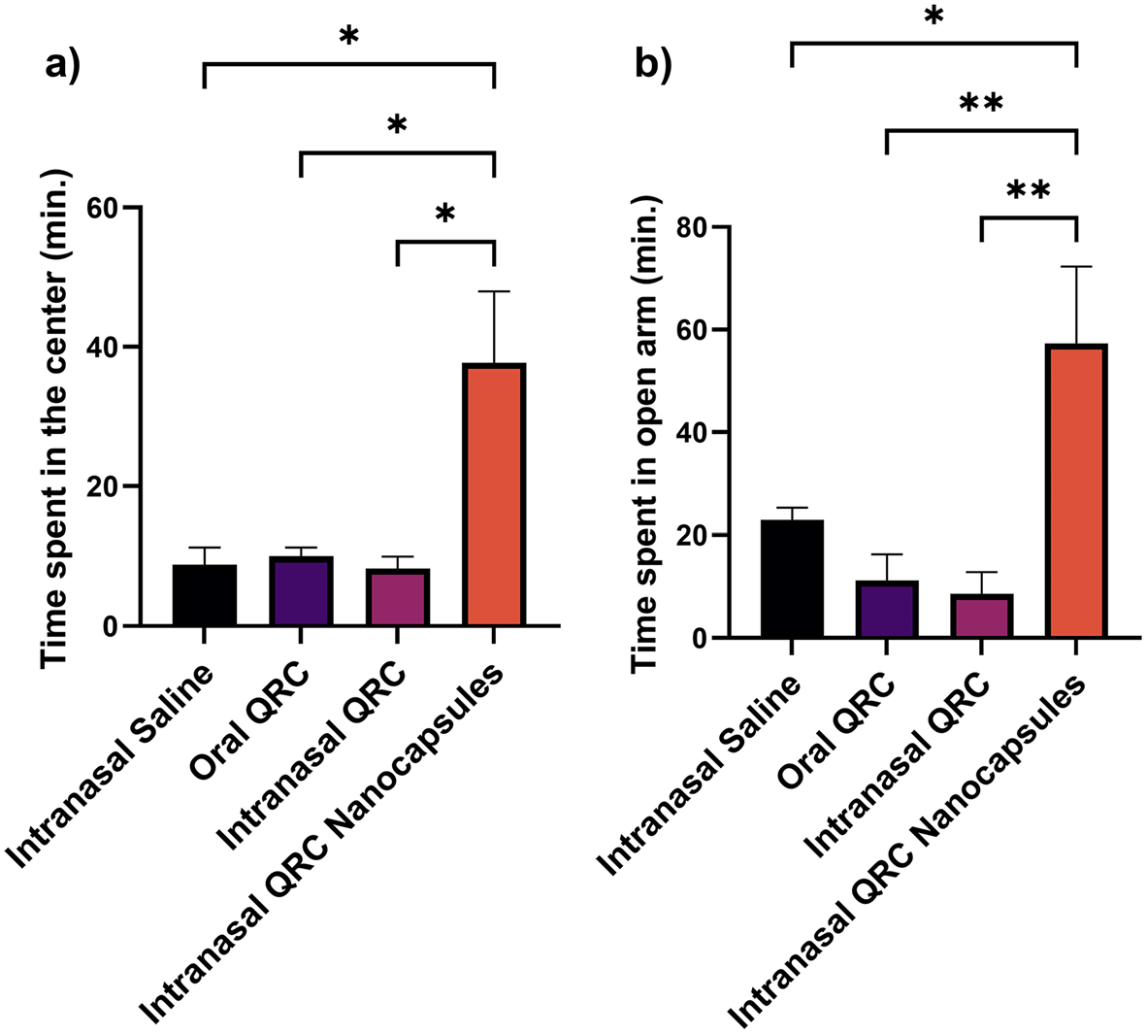
In vivo results of (**a**) time spent in the center of the open field, and (**b**) time spent in the open arm of the elevated plus-maze. The vertical bars represent mean ± Standard Error of Mean (SEM) where values are statistically significant at P < 0.05. *P < 0.05, **P < 0.01.

The anxiolytic effect was assessed by two behavioral assessments which are open field and elevated plus-maze. The results of both assessments are present in (Fig. 6). When analyzing the open field results, intranasal QRC loaded polymeric nanocapsules significantly increased the time spent in the center of the field compared to the control group (P < 0.05), oral QRC dispersion group (P < 0.05), and intranasal QRC dispersion (P < 0.05).

Regarding elevated plus-maze, intranasal QRC loaded polymeric nanocapsules significantly increased the time spent in the open arms compared to the control group (P < 0.05), oral QRC dispersion group (P < 0.01), and intranasal QRC dispersion (P < 0.01).

### Safety studies

In order to ensure the safety of our prepared formulation, safety studies were conducted using both observational nasal irritation test and histopathological examination. Visual observation demonstrated that the animals administered intranasally with both, QRC loaded polymeric nanocapsules as well as QRC dispersion, did not exhibit any signs of nasal mucosal irritation compared to animals administered with intranasal saline. This resulted in classifying both QRC loaded polymeric nanocapsules as well as QRC dispersion in the no irritation level on the nasal mucosa irritation index.

**Figure 7:**
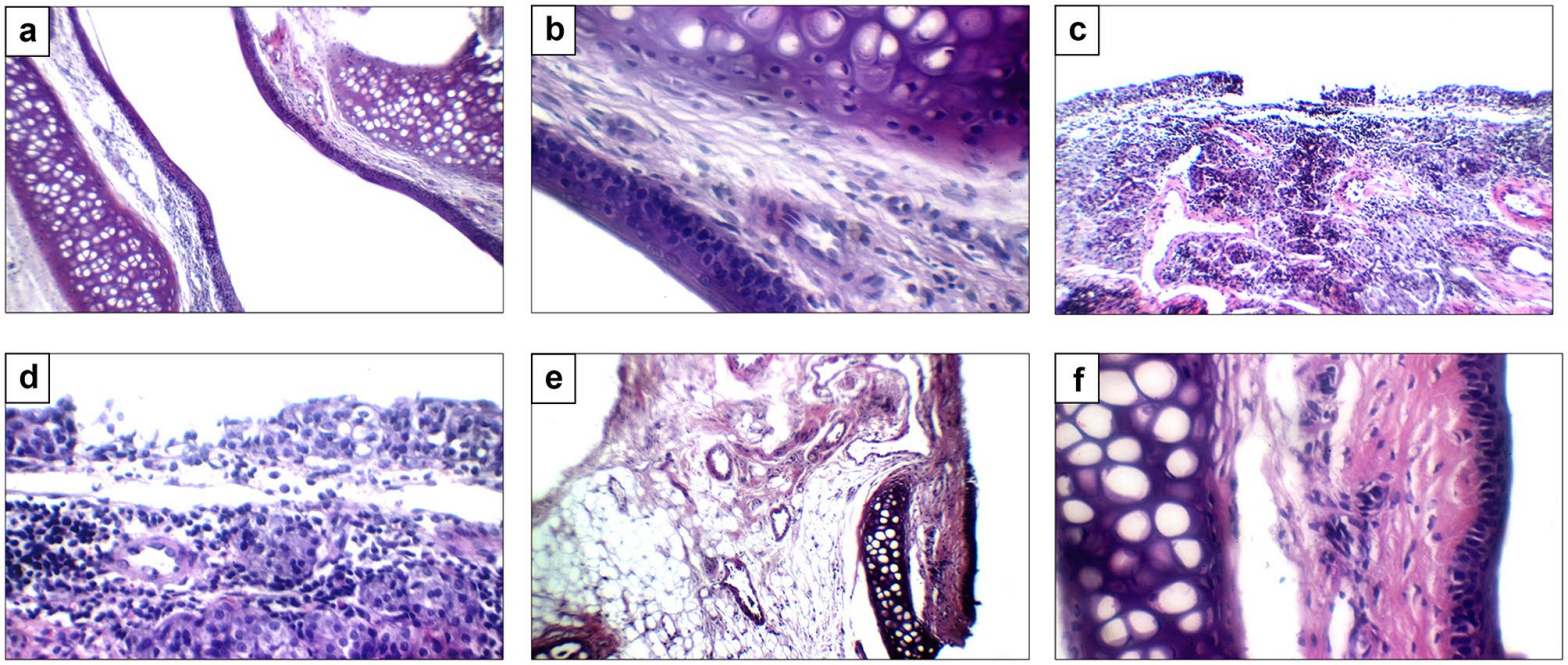
Histopathological examination of nasal mucosa of rats receiving saline at (**a**) × 200 and (**b**) × 400, benzalkonium chloride at (**c**) × 200 and (**d**) × 400, and F3 at (**e**) × 200 and (**f**) × 400.

Histopathological examination of the negative control group showed intact epithelial lining, average submucosa with average blood vessels and cellularity, and average nasal cartilage. While the positive control group demonstrated ulcerated epithelial lining, submucosa with marked hypercellularity, marked inflammatory infiltrate, and marked edema. Regarding our chosen formula, histopathological examination revealed intact epithelial lining, submucosa with average cellularity, average nasal cartilage, and minute edema compared to the positive control group (Fig. 7).

## Discussion

In this study polymeric nanocapsules incorporating QRC were prepared using PCL and Capryol 90. The effect of varying the initial amount of QRC as well as the type of surfactant used was evaluated in order to identify the best formula. The best formula was characterized using multiple characterization techniques. The best formula was also evaluated in vivo using two behavioral tests to assess the anxiolytic effect utilizing the intranasal route.

Nanocapsules are characterized by the presence of an inner core surrounded by an outer polymeric shell. Formulating an efficient drug delivery system depends on the accurate selection of the contributing materials^49^. PCL was chosen as the polymeric outer shell as a result of its advantages including biodegradability, and lack of toxicity^50^. Multiple options exist regarding the choice of an oily core to entrap hydrophobic active ingredients. The most important factor is the solubility of the active pharmaceutical ingredient in the chosen oil as higher solubility results in higher EE%^51^. HSP were used to predict the solubility between QRC and different liquid lipids. HSP are based on the concept that the total cohesive energy can be divided into three main parts. δ_D_ which refers to dispersion forces (van der Waals), δ_P_ that refers dipole forces (polarity), and δ_H_ which refers to hydrogen bonding. The sum of squares of these three parameters results in the square of Hildebrand (total) solubility parameter [Eq. (2)]^52^.

2 $${\delta^{2}}= {\delta^{2}} {\text{D}}+{\delta^{2}}{\text{P}}+{\delta^{2}}{\text{H}}$$

Euclidean distance is used to predict solubility, higher solubility is obtained with smaller distance. The smaller the distance, the higher the affinity between the solute and solvent, hence higher solubility^30^. Among the tested liquid lipids, Capryol 90 exhibited the lowest euclidean distance, thus, the highest solubilizing power for QRC. This inference was validated by previous solubility testing conducted for QRC available in the literature^39,53^ where Capryol 90 (propylene glycol monocaprylate) and propylene glycol displayed the highest solubility for QRC. The solubilization power of Capryol 90 for QRC was also determined experimentally. The high solubility obtained of 28.41 ± 8.91 mg/g indicates the high solubilizing power of Capryol 90. Hence, Capryol 90 was chosen as the liquid lipid.

QRC loaded polymeric nanocapsules were prepared using a modified nanoprecipitation method. In our study, the obtained particle sizes are similar to the particle sizes obtained by dos Santos et al.^54^ and Joo et al.^55^. Changing the amounts of QRC and the type of surfactant used exhibited varying effects of particle size and PDI. When tween 80 was used as a surfactant, the particle size increased from 184.6 to 227.8 nm when QRC amounts changed from 10 to 30 mg. The same effect was observed with poloxamer 188 as the surfactant where the particle size increased from 262.3 with 10 mg QRC to 278.1 nm with 30 mg QRC. These results are in accordance with the results reported by Pegoraro et al*.* where increasing the amount of vitamin E encapsulated in PCL nanocapsules resulted in an increase in the particle size^56^. It is also evident the particle size increased when poloxamer was used instead of tween 80 with both amounts of QRC. It was reported by El-Gogary et al. that tween 80 is characterized by higher adsorption potential compared to poloxamer 407 resulting in smaller particle sizes when tween 80 was used^57^. Regarding PDI, increasing QRC amounts from 10 to 30 mg decreased the homogeneity of sample resulting in higher PDI. Similar observations were reported by Araújo et al. when increasing the amount of cloxacillin benzathine in PCL nanocapsules^58^.

Zeta potential is defined as the effective electric charge on the surface of nanoparticles and is considered one of the main parameters that indicate stability^59^. In our study, all formulations exhibited negative zeta potential values which can be attributed to the dissociation of the carboxylic group of PCL. This dissociation result in the presence of the polar groups of PCL on the surface of the nanocapsules leading to negative zeta potential values^60^. Our prepared formulations exhibited zeta potential values similar to these reported by Zambrano-Zaragoza et al.^61^. The absolute values of zeta potential are expectedly relatively low -despite being in the stable range- as a result of the presence of non-ionic surfactants. It was reported by Sis et al. that the presence of non-ionic surfactants decreases the magnitude of zeta potential on a wide range of pH ranging between 3.0 and 11.0^62^. The other form of stabilization as a result of the presence of non-ionic surfactants is steric stabilization. Steric stabilization is the process by which the adsorbed non-ionic surfactants produce strong repulsion between the particles when the adsorbed layer is hydrated by the molecules of the solvent^43^.

EE% represents the amount of the active constituent encapsulated in the nanoparticles. EE% achieved in our study is similar to the results reported by Ibrahim et al.^63^. The high amount of QRC encapsulated in all the prepared formulations can be attributed to the high solubility of QRC in Capryol 90. It was previously reported that high solubility of the active constituent in oils can result in higher EE%^30^. Upon changing QRC amounts from 10 to 30 mg, the EE% increased from 79.1 and 83.9% to 92.5 and 92.7%. This increase in EE% suggest the high capability of the chosen system -both PCL and Capryol 90- to solubilize QRC in large amounts as it was previously reported that encapsulation efficiency depend on the active constituent miscibility in both polymer and lipid^64,65^. Ersoz et al. reported similar findings were the encapsulation efficiency of QRC increased with increasing QRC initial amount^66^. Among the four prepared formulations, F3 was chosen as the best formulation. This choice was made based on the fact that this formula possessed small particle size, high entrapment efficiency, comparable zeta potential to the remaining formulations, and acceptable PDI. All the following experimental procedure was done on the F3.

The chosen formula exhibited spherical, unilamellar, and well-defined nanoparticles when evaluated using TEM. This was in accordance with what was previously reported by Ibrahim et al.^63^.

In order to analyze the thermal behavior of QRC, PCL, Capryol 90, physical mixture and F3, DSC analysis was conducted. The results have shown the presence of a sharp endothermic peak for both QRC and PCL. When analyzing the results of the physical mixture, it was evident that both PCL and QRC peaks shifted toward lower melting points. The shift in PCL melting point in the physical mixture suggests the homogenous mixing of the polymer and oil^67^.The shift in QRC melting point confirms the ability of Capryol 90 to solubilize QRC which resulted in an earlier QRC melting. The chosen formula demonstrated a peak shift from 58.66 to 46.37 °C which suggests a closer component interaction because of the formation of nanoparticles. Also, the QRC peak disappeared, and a new broad peak appeared. This disappearance indicates successful encapsulation of the active ingredient into nanoparticles^68,69^. These results are in accordance with what was previously reported by Penteado et al. where the melting peak of perillyl alcohol disappeared after its incorporation in chitosan coated PCL nanocapsules^70^.

The nature of the structure of QRC, PCL, physical mixture and F3 whether crystalline or amorphous was analyzed using XRD. QRC exhibited highly crystalline structure while PCL demonstrated semi-crystalline structure. The mixture of PCL and QRC demonstrated the characteristic peaks of both. However, there was a complete disappearance of QRC peaks in F3. This can be explained by a change in the structure of QRC from crystalline to amorphous during the nanocapsules preparation. This indicates the incorporation of QRC in the prepared nanoparticles^71^. Similar findings were reported by Carletto et al. when resveratrol peaks disappeared after its incorporation in PCL nanocapsules^72^.

QRC loaded polymeric nanocapsules were able to provide much lower release for QRC compared to free QRC while modifying the release kinetics of QRC. The prepared polymeric nanocapsules were able to modify the release kinetics of QRC from first order model to Higuchi model as well as the n value of Korsmeyer–Peppas model from 0.316 to 0.48. The release pattern of QRC loaded polymeric nanocapsules exhibited biphasic pattern with initial burst release followed by a sustained release pattern. This pattern was previously reported when preparing polymeric nanoparticles using nanoprecipitation method^73^. The initial burst release can be attributed to the desorption of the active ingredient from the surface of the nanocapsules and/or the release of active molecules present near the surface of the nanocapsules. The second sustained release occur as a result of the active ingredient diffusion through the nanocapsules and erosion of the polymeric shell^74^. This was further confirmed by the release kinetics analysis. It was determined that Higuchi’s diffusion model is the release kinetics model of the prepared QRC loaded polymeric nanocapsules since Higuchi model had the highest adjusted r^2^ value. Regarding Korsmeyer–Peppas model, it was previously reported that in case of spherical geometry, n value between 0.43 and 0.85 indicates that the release mechanism is governed by both diffusion and polymer erosion mechanisms^75^. The n value of our formulation was 0.48 indicating QRC release by both mechanisms as previously mentioned. This may indicate that QRC diffuse through the prepared nanocapsules after the degradation of thin polymeric outer layer. This was in accordance with what was previously mentioned by Abdel-Rashid et al.^76^. It is also worth mentioning that both QRC loaded polymeric nanocapsules and QRC dispersion almost reached complete release at the end of the experiment indicate the successful attainment of the sink condition and the availability of the active ingredient transfer to the release media without limitations^77^.

The next step is the in vivo evaluation of the chosen formula administered intranasally. Behavioral assessment was used to assess the anxiolytic effect of QRC loaded polymeric nanocapsules compared to control group, oral QRC dispersion and intranasal QRC dispersion. Two behavioral assessments were used as anxiety models which are open field and elevated plus-maze. Rats placed in foreign environment (open field and elevated plus-maze) either spend time in relative safety (near the walls of the open field, and in the closed arms of the elevated plus-maze) or explore dangerous area (center of the open field, and in the open arms of the plus-maze). Anxiolytic activity is determined by higher tendency of the rats to explore dangerous areas^78^.

The highest anxiolytic effect obtained from behavioral testing was achieved when the intranasal route was utilized in administering the prepared QRC loaded polymeric nanocapsules. All the parameters analyzed in both behavioral assessments indicated that intranasal QRC loaded polymeric nanocapsules significantly improved the assessed parameters resulted in the best anxiolytic effect. These results are in accordance with what was previously reported by Priprem et al.^17^.

Improvements in QRC anxiolytic effects observed with the encapsulation of QRC in polymeric nanocapsules administered intranasally is most likely due to the improved QRC delivery to the brain which can be attributed to the ability of nanoparticles to directly deliver therapeutics to the brain when administered intranasally. Although this inference is well supported in the available literature^79–81^, further validation might be required to confirm the improved accumulation of QRC in the brain experimentally.

Ensuring the safety of any prepared formulation is paramount in any pharmaceutical development process. Our prepared formulation as well as QRC dispersion demonstrated no signs of nasal irritation when administered intranasally compared to when saline was administered intranasally. Based on these observations, both QRC loaded polymeric nanocapsules as well as QRC dispersion were placed in the no irritation level on the nasal mucosa irritation index indicating that both are cilio-friendly^36^. To further validate the safety of our chosen formula, histopathological examination was conducted to analyze the rats’ mucosa when the rats received either saline, benzalkonium chloride, or the chosen formula. Compared to rats receiving benzalkonium chloride, the rats that received our chosen formula demonstrated normal nasal mucosal structure with minute edema compared to rats receiving benzalkonium chloride indicating better tolerability and the safety of the prepared formulation.

## Conclusion

The present study successfully formulated QRC loaded polymeric nanocapsules using nanoprecipitation method of preparation. Both the type of surfactant and the drug amount were used with different levels. It was evident that tween 80 produced particles with smaller size compared to these produced by poloxamer 188. It was also evident that increasing the drug amount increased the particle size as well as the EE%. The best formula was chosen and assessed for its anxiolytic effect in vivo using two behavioral tests after intranasal administration which demonstrated significant improvements in the anxiolytic effect of QRC. This improvement was achieved while showing good safety and tolerability profile. The obtained results proved that encapsulating QRC in polymeric nanocapsules to be administered intranasally is a promising and successful strategy to improve the anxiolytic effect of QRC.

## Data Availability

The datasets used and/or analyzed during the current study are available from the corresponding author on reasonable request.

## Abbreviations

APA: American Psychological Association
BBB: Blood–brain barrier
DSC: Differential scanning calorimetry
EE: Encapsulation efficiency
GABA: γ-Aminobutyric acid
GBD: Global burden of diseases, injuries, and risk factors study
H&E: Hematoxylin and eosin
HSP: Hansen solubility parameters
HSPiP: Hansen solubility parameters in practice
PCL: Polycaprolactone
PDI: Polydispersity Index
QRC: Quercetin
SD: Standard deviation
SEM: Standard error of mean
TEM: Transmission electron microscopy
XRD: X-Ray diffraction
